# Long-Term Clinical Efficacy and Perioperative Safety of Endoscopic Submucosal Dissection versus Endoscopic Mucosal Resection for Early Gastric Cancer: An Updated Meta-Analysis

**DOI:** 10.1155/2018/3152346

**Published:** 2018-01-11

**Authors:** Yajie Zhao, Chengfeng Wang

**Affiliations:** Department of Pancreatic and Gastric Surgery, National Cancer Center/Cancer Hospital, Chinese Academy of Medical Sciences and Peking Union Medical College, Beijing 100021, China

## Abstract

**Background:**

To systematically evaluate the safety and efficacy of endoscopic submucosal dissection (ESD) versus endoscopic mucosal resection (EMR) for early gastric cancer (EGC).

**Methods:**

We searched the databases of PubMed, Web of Science, EMBASE, and the Cochrane Library from January 2000 to April 2017 and included studies that compared the outcomes of ESD with EMR for EGC. These eligible studies that met the inclusion criteria were screened out and were assessed by two independent investigators.

**Result:**

In total, 18 retrospective cohort studies were eligible for analysis. Our results indicated that ESD is more beneficial than EMR in increasing the complete resection rate and en bloc resection rate and decreasing the local recurrence rate. However, ESD prolonged operative time and increased incidence of gastric perforation than EMR. No differences were found in postoperative bleeding rate between the two approaches.

**Conclusion:**

Compared with EMR, ESD offers higher complete resection rate, higher en bloc resection rate, and lower local recurrence rate but has prolonged operative time and increased incidence of gastric perfusion. There is no statistical difference in the rate of postoperative bleeding between the two groups. However, the above conclusion needs further verification by well-designed, randomized trials with larger samples and long follow-up periods.

## 1. Introduction

Endoscopic mucosal resection (EMR) technique is widely accepted as standard treatment for early gastric cancer (EGC) [1]. It declares that the technique of endoscopic mucosal resection is indicated for early gastric cancer with no lymph node metastasis. EMR is widely accepted by endoscopists for its advantages of being minimally invasive, cost effective, and well tolerated and offering good quality of postoperative life [2, 3]. Despite the convenience of EMR, larger lesions cannot be completely removed by EMR in one attempt; therefore, the entire pathologic specimen cannot be retrieved, and a proper treatment decision cannot be made by clinicians through precise pathological examination, which leads to a potentially high risk of local tumor recurrence or excessive treatment. To overcome the disadvantages of EMR, ESD is used for the resection of large lesions [4]. ESD technology can directly remove tumors from the submucosal layer. However, owing to larger wound size and difficulty in performing the technique, ESD has higher incidence of postoperative complications such as postoperative bleeding and perforation. Some studies have compared the application of EMR and ESD, but with inconsistent results. There are few meta-analyses comparing the efficacy and safety of endoscopic submucosal dissection with endoscopic mucosal resection for EGC. Therefore, we performed a meta-analysis to assess the efficacy and safety of ESD and EMR in EGC and provide clinical evidence for endoscopic treatment of early gastric cancer.

## 2. Materials and Methods

### 2.1. Literature Search

**Figure 1:**
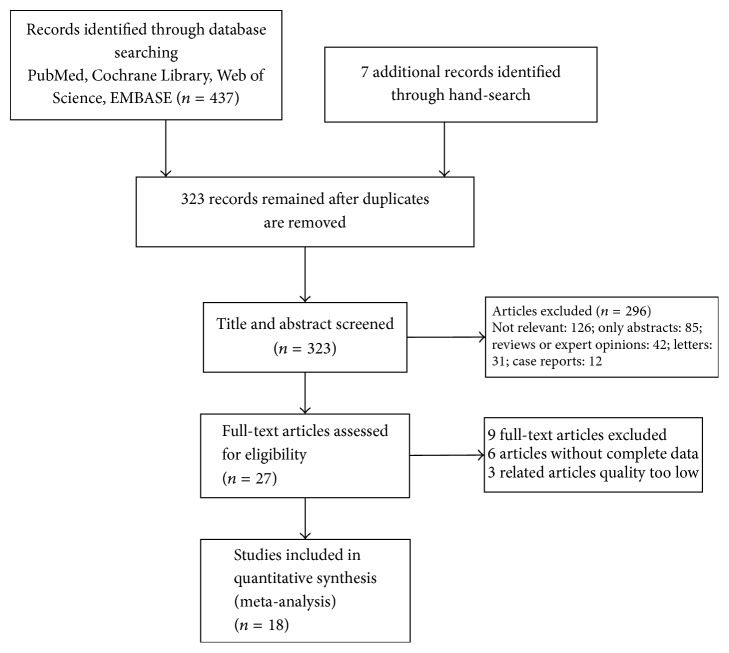
Flow diagram.

We searched the databases of Web of Science, PubMed, EMBASE, and the Cochrane Library for journal articles published from January 2000 to April 2017. The following search terms were used: “ESD” or “endoscopic mucosal resection” and “endoscopic mucosal resection” or “EMR” and “early gastric cancer” or “EGC”. Both free terms and MeSH words were included. There was no language restriction and two independent researchers performed this search. Final inclusion was determined by consensus. The results of the search strategy are shown in Figure 1.

### 2.2. Inclusion Criteria

The following studies were included: (1) those in which the included patients were diagnosed with EGC based on histology; (2) studies that were conducted to compare ESD and EMR for EGC; (3) those where the endpoints included therapeutic effect index and postoperative complications; and (4) those where if the same data had been published multiple times, the latest publication was considered.

### 2.3. Exclusion Criteria

The following studies were excluded: (1) those in which the detailed surgical type was not reported; (2) those that had participants without early gastric cancer, instead with adenoma, precancerous lesions, or other gastric lesions; (3) studies referring to recurrent early gastric cancer; (4) those that had no data regarding therapeutic effects or complications and those in which the study outcomes did not include complete or available perioperative outcomes and postoperative data; (5) those which reported data used in a later study; and (6) case reports, abstracts, letters, comments and reviews or guideline articles without original data, and studies that presented insufficient data.

### 2.4. Data Extraction

The following detailed data were extracted by the two independent investigators: authors; year of publication; country; study design; surgery type; number of patients; and the following clinical data: (1) operation time: the time from marking to complete removal of the tumor and including the time for hemostasis; (2) en bloc resection: removing the tumor in one piece without fragmentation; (3) complete resection: the histologic examination shows the lateral margins being tumor-free ⩾ 2 mm and the vertical margins being tumor-free *⩾* 0.5 mm; (4) postoperative bleeding: postoperative hematemesis or melena needs an endoscopic hemostatic procedure; (5) perforation: free air was seen on abdominal radiograph or endoscopic observation of mesenteric fat after the operation. (6) Local recurrence: the same histological type of cancer was found at the resection site more than 6 months after the operation.

#### 2.4.1. Statistical Analysis

Meta-analysis was conducted with Review Manager (version 5.3.0) software. Odds Ratios (ORs) were used to analyze the dichotomous variables and 95% confidence interval (CI) values were reported. The Mantel-Haenszel, Chi-square, and *I*^2^ tests were used to test the heterogeneity between studies. If *I*^2^ > 50%, this suggested significant heterogeneity; a random effects model was applied. If *I*^2^ < 50%, this suggested not significant heterogeneity; a fixed effects model was applied. If *P* < 0.05, this considered statistical significance. Funnel plots were used to evaluate potential publication bias.

#### 2.4.2. Characteristics of the Included Studies and Quality Assessment

18 retrospective cohort studies were included in this meta-analysis. The total included patients were 7395, of whom 3596 were EMR group and 3799 were ESD group. The detailed characteristics of all the included studies are shown in Table 1. The observational clinical studies (OCS) were scored based on the Newcastle-Ottawa Scale (NOS) System that included assessments of selection, comparability, and exposure or outcome. Each study was given score of 9 in total; if the total score was ≥7, the OCS was considered to be of high quality.

## 3. Meta-Analysis Results

### 3.1. Operation Time

**Figure 2:**
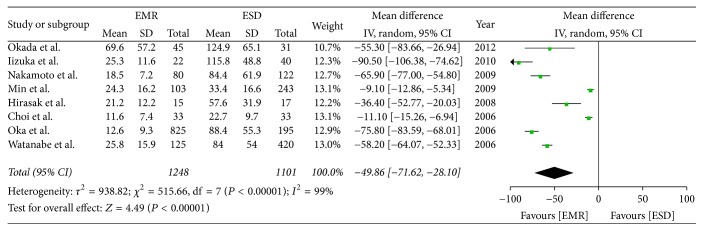
Meta-analysis of operation time.

Eight studies reported the operation time. The result showed that the ESD group was associated with longer operative times than the EMR group (OR = −49.86; 95% CI, −71.62–−28.10; *P* < 0.00001; *I*^2^ = 99%); a random effect model was applied (Figure 2).

### 3.2. En Bloc Resection Rate

**Figure 3:**
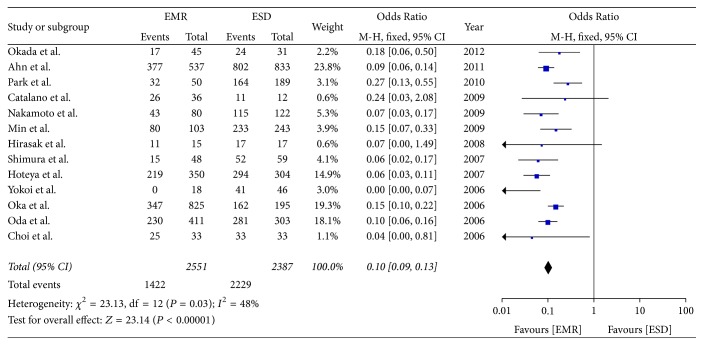
Meta-analysis of en bloc resection rate.

Thirteen studies reported on the en bloc resection rate. The analysis showed a higher rate of en bloc resection in the ESD group than in the EMR group (OR = 0.10; 95% CI, 0.09–0.13; *P* < 0.00001, *I*^2^ = 48%); hence, a fixed effect model was applied (Figure 3).

### 3.3. Complete Resection Rate

**Figure 4:**
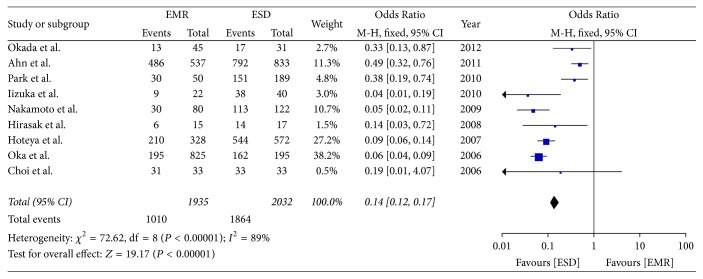
Meta-analysis of complete resection rate.

Nine studies reported on the complete resection rate. The meta-analysis showed the rate of complete resection was higher in the ESD group than in the EMR group (OR = 0.14; 95% CI, 0.07–0.29; *P* < 0.00001; *I*^2^ = 89%); hence, a random effect model was applied (Figure 4).

### 3.4. Postoperative Bleeding

**Figure 5:**
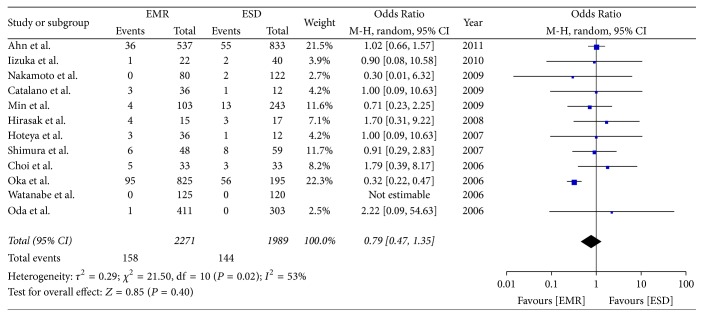
Meta-analysis of postoperative bleeding.

Twelve included studies reported on postoperative bleeding. No statistical difference was seen with respect to postoperative bleeding rates between the two groups (OR = 0.79; 95% CI, 0.47–1.35; *P* = 0.40; *I*^2^ = 53%); hence, a random effect model was applied (Figure 5).

### 3.5. Incidence of Perforation

**Figure 6:**
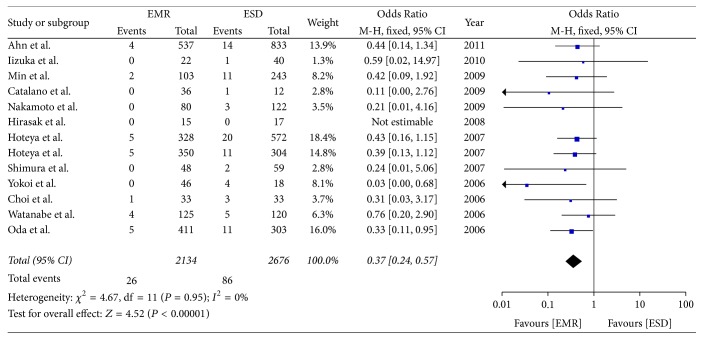
Meta-analysis of incidence of perforation.

Thirteen included studies reported on the incidence of perforation. The meta-analysis showed that the incidence of perforation was higher in the ESD group than EMR group (OR = 0.37; 95% CI, 0.24–0.57; *P* < 0.00001; *I*^2^ = 0%); hence, a fixed effect model was applied. There was a significant difference between the two groups (Figure 6).

### 3.6. Local Recurrence Rate

**Figure 7:**
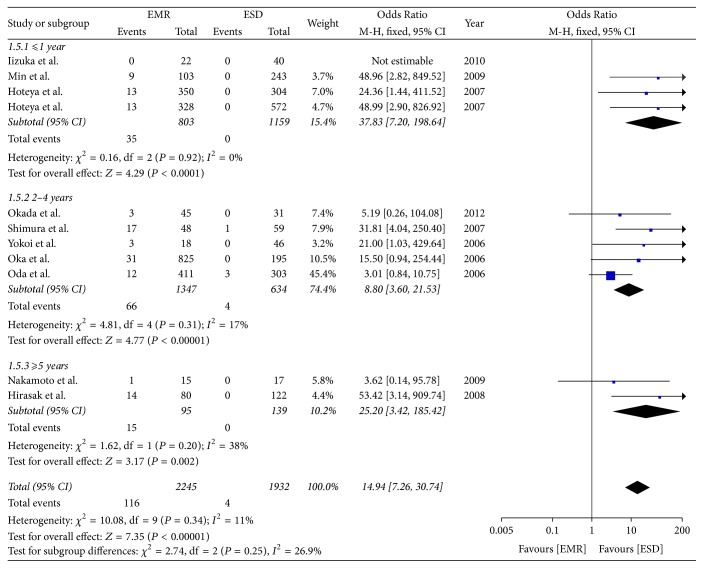
Meta-analysis of local recurrence rate.

Four studies compared the local recurrence rate of postoperative time ⩽ 1 year. The meta-analysis showed the rate of local recurrence in the ESD group was lower than in the EMR group (OR = 37.83; 95% CI, 7.20–198.64; *P* < 0.0001; *I*^2^ = 0%). Five studies compared the recurrence rate of postoperative time > 2 years but <4 years and found that the local recurrence rate in the ESD group was lower than in the EMR group (OR = 8.80; 95% CI, 3.60–21.53; *P* < 0.00001; *I*^2^ = 17%). Two studies compared the recurrence rate of postoperative time *⩾* 5 years and found that the rate was lower in the ESD group than in the EMR group (OR = 25.20; 95% CI, 3.42–185.42; *P* = 0.002; *I*^2^ = 38%); a fixed effect model was applied (Figure 7).

## 4. Subgroup of Meta-Analysis

### 4.1. Subgroup Analysis of the En Bloc Rate

**Figure 8:**
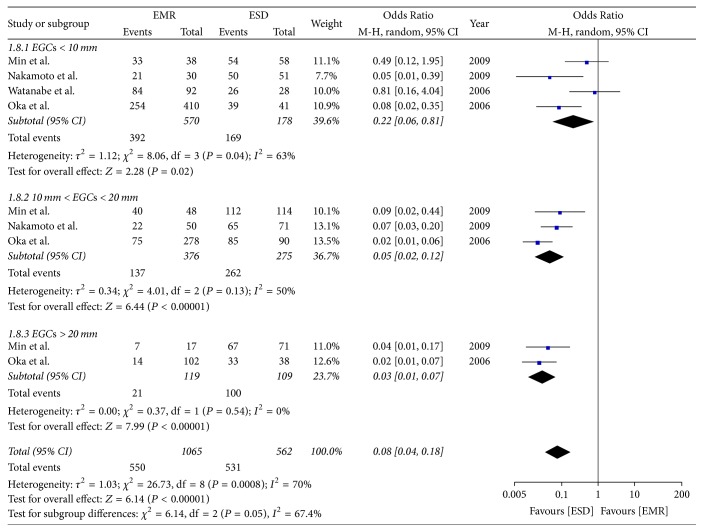
Subgroup analysis of the en bloc rate.

Four studies compared the rate of en bloc for lesions < 10 mm. The meta-analysis showed the rate of en bloc for lesions < 10 mm in the ESD group was higher than in the EMR group (OR = 0.22; 95% CI, 0.06–0.81; *P* = 0.02; *I*^2^ = 63%). Three studies compared the en bloc rate for lesions > 10 mm but <20 mm and found that the rate of en bloc in the ESD group was higher than in the EMR group (OR = 0.05; 95% CI, 0.02–0.12; *P* < 0.00001; *I*^2^ = 50%). Two studies compared the en bloc rate for lesions > 20 mm and found that it was higher in the ESD group than in the EMR group (OR = 0.03; 95% CI, 0.01–0.07; *P* < 0.00001; *I*^2^ = 0%); a random effect model was applied (Figure 8).

### 4.2. Subgroup Analysis of Complete Resection Rate

**Figure 9:**
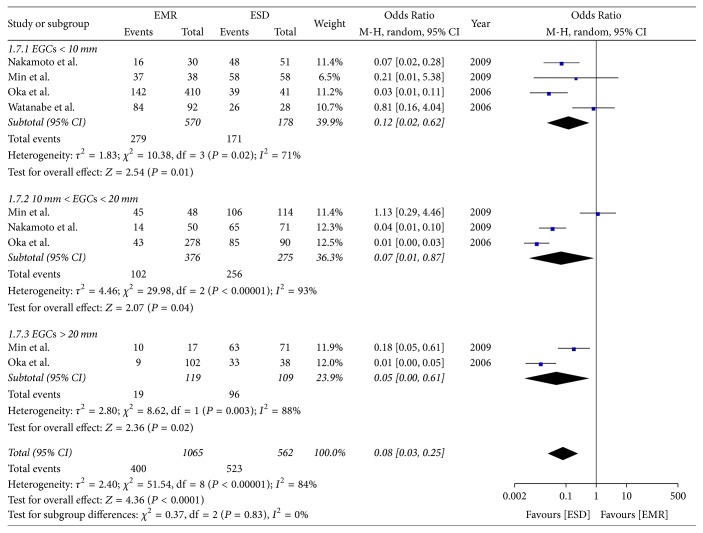
Subgroup analysis of complete resection rate.

Four studies compared the complete resection rate for lesions < 10 mm. The meta-analysis showed the rate of complete resection in the ESD group was higher than in the EMR group (OR = 0.12; 95% CI, 0.02–0.62; *P* = 0.01; *I*^2^ = 71%). Three studies compared the rate of complete resection for lesions > 10 mm but <20 mm and found that the rate in the ESD group was higher than in the EMR group (OR = 0.07; 95% CI, 0.01–0.87; *P* = 0.04; *I*^2^ = 93%). Two studies compared the rate of complete resection for lesions > 20 mm and showed the rate of complete resection was higher in the ESD group than in the EMR group (OR = 0.05; 95% CI, 0.00–0.61; *P* = 0.02; *I*^2^ = 88%); a random effect model was applied (Figure 9).

## 5. Publication Bias

**Figure 10:**
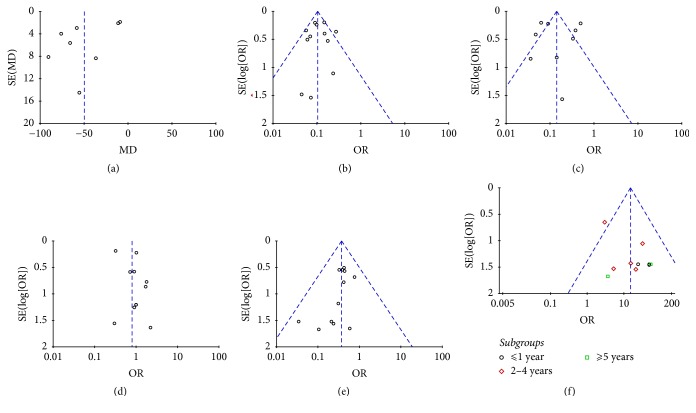
Funnel plots were created to assess the publication bias in our meta-analysis. In the absence of publication bias, it assumes that studies with high precision will be plotted near the average, and studies with low precision will be spread evenly on both sides of the average, creating a roughly funnel-shaped distribution. (a) Operation time. (b) En bloc resection rate. (c) Complete resection rate. (d) Postoperative bleeding. (e) Incidence of perforation. (f) Recurrence rate.

Deviation from this shape can indicate publication bias. There was no evident asymmetry in the funnel plots (Figure 10), suggesting a low probability of publication bias.

## 6. Discussion

EMR is widely used treatment for early gastric cancer. However, this kind of technique is with a high local recurrence rate for incomplete resection. In order to overcome this problem, endoscopic submucosal dissection was developed to resected larger lesions that could not be removed using the EMR technique. Although ESD is a new and exciting technology, the technique of ESD is difficult and needs to acquire skills in manipulating treatment devices. Therefore, a large learning gap exists among different endoscopists. What is more, the cost of ESD is higher. The meta-analysis showed longer operation time in ESD group than in the EMR group. ESD is technically difficult and time-consuming mainly because of complex procedures. Intraoperative bleeding sometimes prolongs the time of operation, although bleeding during the operation is sometimes inevitable. Effectively controlling intraoperative bleeding and reducing intraoperative bleeding are the biggest challenge. With growing skill and experience, the operation time of ESD may be reduced.

Postoperative bleeding is a common complication of endoscopic therapy. The results of previous studies have indicated that the rate of postoperative bleeding in ESD is higher than that of EMR, and the reported postoperative bleeding rates varied across studies, although this meta-analysis showed that there was no significant difference in postoperative bleeding rate between the two groups. Perforation is another common complication of endoscopic treatment. It may be related to the size of the lesion or the ulceration. In general, lesion size > 3 cm, ulceration, and unskillful operation increase the risk of perforation. This result of this meta-analysis showed that the rate of perforation was higher in the ESD group. In most cases, the perforation was small and did not need surgical treatment. With the development of technologies, the procedural bleeding and perforation may be reduced.

ESD showed advantages regarding effect outcomes. This meta-analysis showed higher rate of en bloc resection and complete resection in ESD group than in EMR group. As the complete resection rate and en bloc resection rate were limited to the lesion size, we performed subgroup analysis according to the tumor size in order to decrease the heterogeneity, This subgroup analysis showed a superior complete resection rate and en bloc resection rate in the ESD group not only for lesions > 10 mm, <20 mm, and > 20 mm, but also for the lesions < 10 mm. En bloc resection will have technical advantage in procuring the entire pathologic specimen and providing accurate histopathologic evaluation, making it possible to increase the rate of complete resection. Complete resection is beneficial for achieving a negative tumor margins. Therefore, ESD has a technical advantage in achieving a negative tumor margin and reducing the local recurrence rate. The results of this meta-analysis also show the lower rate of local recurrence in the ESD group than EMR group in postoperative time ≤ 1-year, >2-year, <4-year, and ≥5-year subgroup.

There are several limitations in this meta-analysis. First, this meta-analysis included only a single western study from Italy. Therefore, the conclusion may not apply in western countries. Second, all included studies in this analysis are observational clinical studies, which may have affected the results. Finally, not all studies provide clear definitions or criteria for any project, so the outcome may be more or less affected. Another potential limitation is that operation experience and methods used at different hospitals and specialist centers could have produced different outcomes and increased the heterogeneity among the included studies.

## 7. Conclusions

ESD showed advantages compared with EMR regarding the high rate of en bloc resection and complete resection and low local recurrence rate, but also having higher rates of perforation and extended operation time; the perforation was usually small and having surgical treatment was not necessary. The results should be confirmed by large samples and randomized trials from different regions of the world.

## Figures and Tables

**Table 1:** The characteristics of all the included studies.

Author	Year	Country	Study type	Group	Patients Number	Study quality
Tanabe et al. [5]	2014	Japan	Retro	EMR	359	5
				ESD	421	
Okada et al. [6]	2012	Korea	Retro	EMR	45	5
				ESD	31	
Ahn et al. [7]	2011	Korea	Retro	EMR	537	5
				ESD	833	
Park et al. [8]	2010	Korea	Retro	EMR	50	5
				ESD	189	
Watanabe et al. [9]	2006	Japan	Retro	EMR	146	7
				ESD	219	
Shimura et al. [10]	2007	Japan	Retro	EMR	22	5
				ESD	40	
Nakamoto et al. [11]	2009	Japan	Retro	EMR	80	5
				ESD	122	
Catalano et al. [12]	2009	Italy	Retro	EMR	36	7
				ESD	12	
Min et al. [13]	2009	Korea	Retro	EMR	103	7
				ESD	243	
Hoteya et al. [14]	2009	Japan	Retro	EMR	328	7
				ESD	572	
Shimura et al. [10]	2007	Japan	Retro	EMR	48	9
				ESD	59	
Hoteya et al. [15]	2007	Japan	Retro	EMR	350	7
				ESD	304	
Oda et al. [16]	2006	Japan	Retro	EMR	411	7
				ESD	303	
Oka et al. [17]	2006	Japan	Retro	EMR	825	9
				ESD	195	
Choi et al. [18]	2006	Japan	Retro	EMR	33	7
				ESD	33	
Watanabe et al. [9]	2006	Japan	Retro	EMR	125	7
				ESD	120	
Odashima et al. [19]	2006	Japan	Retro	EMR	80	7
				ESD	57	
Yokoi et al. [20]	2006	Japan	Retro	EMR	18	7
				ESD	46	

*EMR* = endoscopic mucosal resection, *ESD* = endoscopic submucosal dissection, and *the Newcastle-Ottawa System*: the quality of the nonrandomized studies was assessed by using this system, and the quality of the studies was evaluated by examining three items: patient selection, comparability of groups, and assessment of outcome.
